# Is the profitability of Islamic and conventional banks driven by the same factors?—A study of banking in the Middle East

**DOI:** 10.1371/journal.pone.0289264

**Published:** 2023-08-07

**Authors:** Iwona Sobol, Łukasz Dopierała, Przemysław Wysiński

**Affiliations:** Faculty of Economics, Department of International Business, Division of International Financial Markets, University of Gdansk, Gdansk, Poland; Usak University: Usak Universitesi, TURKEY

## Abstract

The purpose of this paper is to contribute to the existing literature by investigating the determinants of the profitability of Islamic and conventional banks in the Middle East region and revealing the most important factors for these two types of banks. Few papers have studied the performance of Islamic banks and compared their performance with conventional banks. The results from these limited research papers are also various, mainly because the sample sizes are small, or they have analysed data only from one country. Our research used a fixed effect panel data analysis on a sample of 270 banks (111 Islamic and 159 conventional banks) from 12 Middle East countries. We used an unbalanced annual panel of data covering the period 2012–2020. The results show that bank size, equity to assets, annual GDP growth, and annual average oil price have a significant positive effect on Islamic banks’ profitability, while non-performing loans to total gross loans and cost of running operations to operating income have a significant negative effect on both bank types. The results also show that non-performing loans to total gross loans and annual GDP growth contribute more to conventional banks profitability, while oil price contributes only to Islamic banks performance. Inflation and net loans to total assets have no effect on bank profitability for either Islamic or conventional banks. Furthermore, we also found that the Islamic banking industry had a more competitive structure. Our findings have important implications for managers, policy makers, investors and other stakeholders. They can help them to make decisions regarding investments, plans, budgeting, evaluation and the management of business operations.

## 1. Introduction

A well-functioning banking system is vital for any economy. By attracting money from depositors and directing it towards investors, banks boost the economy’s overall wealth. Profitable banks contribute to better resource allocation and economic growth. They promote it by providing finance for worthwhile projects [1]. Trujillo-Ponce [2] pointed out that they are able to infuse money into the economy by providing loans, which is crucial for the stability of the banking system. A similar opinion was given by Rashid and Jabeen [3] who maintained that a profitable banking sector provides a safeguard against negative shocks, and helps to stabilise and strengthen the financial system, while inefficient banking has a negative impact on the stability of the financial system and on economic growth. There is empirical evidence supporting the thesis that profitable banks are less likely to fail [4]. Therefore, bank profitability is considered one of the key indicators for forecasting bank failures, using measures such as the Z-Score and the CAMELS rating system [5]. Furthermore, according to Bayar et al. [6], a well developed and stable banking sector may have a positive impact on the health of the population. Thus, studies on bank profitability and its determinants have not only theoretical, but also empirical significance. Their results can help to make crucial decisions by policy makers, regulators, bank managers, investors and customers.

The financial world has established conventional banking as a standard practice that is primarily motivated by the profit maximisation concept [7]. However, since the 1970s, there have been Islamic banks operating in the world, alongside conventional institutions. Islamic banks should perform not only the same functions as their conventional counterparts but in addition, as a renowned Muslim economist, Muhammad Umer Chapra, states they should contribute richly to the achievement of the major socio-economic goals of Islam [8]. Those goals include economic prosperity with full employment and an optimum rate of economic growth, socio-economic justice, and equitable distribution of wealth and monetary stability. Thus, Islamic banks should act ethically and care about the well-being of their society. In order to ensure this ethicality, they need to conduct operations in accordance with the principles of sharia, which is the religious law of Muslims. The basic sharia principle applied by Islamic financial institutions is the prohibition of usury (Arab. riba). This principle has its origins in the holy book of Muslims–the Koran. The hadiths, which describe the life and actions of Muhammad, the Messenger of Allah, also state that riba is condemned. However, neither the Koran nor the hadiths define what riba is; nevertheless, according to most Islamic economists, riba is any sort of increase over the principal amount [9]. So, it has a different meaning than in contemporary Western civilization, where usury is defined as the practice of charging excessive, unreasonably high interests on loans. The prohibition of riba has huge implications on operations conducted by Islamic banks since none of them can be based on interest. Other principles which should be obeyed by Islamic financial institutions include the avoidance of uncertainty (gharar), prohibition of speculation (maysir), prohibition of trading in illegal (haram) products (e.g., alcohol, pork, pornography, tobacco), and reliance on the participation model of banking [10]. The compliance of practices and activities (e.g., products, instruments, operations, and management) of the Islamic bank with the above-mentioned principles is supervised by the sharia supervisory board (SBB) [11]. Islamic banks need the approval of a SSB in every aspect of their operations [12].

Given the fact that Islamic banks need to comply with the sharia principles, the question arises whether they can achieve the same profitability as conventional banks and whether their profitability is driven by the same factors. Thus, the main purpose of this paper is to contribute to the existing literature by investigating the determinants of the performance of Islamic and conventional banks and revealing the most important factors for these two types of banks.

This study focuses on the countries of the Middle East. In this region of the world both Islamic and conventional banks operate, which allows us to examine and compare the determinants of profitability for the two types of banking. Moreover, the Middle East is the region of the world with the most developed Islamic banking. Thus, it may indicate that Islamic banks are competitive with their conventional counterparts.

Both Islamic and conventional banks are profit-maximising organisations that are essential for the effective use of resources and, as a result, lessen information asymmetries, assist in lowering transaction costs, and facilitate diversification for small investors and savers. As financial intermediaries, they provide services such as: asset transformation, a payment system, custodial services, and risk management. In theory, these two types of banks differ significantly. While conventional banks’ intermediation is primarily debt-based and permits risk transfer, Islamic banks’ intermediation is asset-based and focuses on risk sharing [13]. The Islamic banking model enables the risk to be shifted from assets to the liabilities side, which limits the impact of adverse shocks on their solvency and positively affects performance. On the other hand, the increased loyalty of religious customers could ease the pressure on Islamic banks to perform [14]. Also, there is an additional body in Islamic banks, the sharia supervisory board, which could affect profitability in the case of the expenses level.

There might be fundamental differences between the Islamic and conventional banking in theory; in practice, however, they are relatively small. Principally, Islamic banks should rely on the participation model, where profits and losses are shared between the contracted parties. However, the competition has forced them to rely mainly on fixed return products [15], where the markups are often strongly correlated with the interest rates observed in the conventional banking.

It is difficult to predict whether the performance of Islamic banks is affected by the same determinants as the conventional institutions. On the one hand, due to the specific Islamic activity, the profitability of Islamic banks would not be determined by the same factors that have an impact on conventional banks. Looking at the issue on the other hand, the similarity of Islamic banking practice to the conventional banking model may result in the observation that there is no difference between the profitability determinants for Islamic and conventional banks. Our research will give an answer to this issue.

Our paper seeks to make the following contributions to the literature on the determinants of banks’ performance. First, to the best of our knowledge, this study, along with those by Mateev et al. [16,17], represents one of the few analyses on the determinants of Islamic and conventional bank performance that utilise such a large sample. It takes into account 159 conventional and 111 Islamic banks from 12 countries, which makes our data base unique. Other studies that included Islamic banks are either based on data from one country (e.g., [18–22]) or the number of banks, which they examine, is much smaller (e.g., [23–25]). We have started with an unbalanced panel of 237 conventional banks and 121 Islamic banks. Unfortunately for some banks data were not available for more than one year and such kinds of bank observations were excluded. While there is clearly a difference between the number of Islamic and conventional banks, still nearly 41% of our observations relate to Islamic banks. This is higher than in the most previous studies, which were based on less balanced databases with a greater share of conventional banks. Our study is based on the most recent data available, including the first year of the Covid-19 pandemic. The period of the study (9 years) is long enough to allow us to reach reliable results and draw consistent conclusions.

Second, we contribute to the existing empirical studies on the determinants of profitability of both conventional and Islamic banks, which have yielded ambigous findings. Thus, our study gives information whether the same determinants impact the performance of the two types of banking. This issue is especially important for bank managers, investors and policy makers, who should receive the answer for the question whether Islamic banks should have the same regulations to abide by as the conventional institutions.

Third, we used static and dynamic models which suggest that in the case of the conventional banks there is a much stronger ROAA persistence. So we found that the Islamic banking industry had a more competitive structure. In addition, we included in the static approach the estimation of fixed effect models and random effect models as well as subsample analysis and alternative independent variables to ensure the robustness of the results.

Our study provides detailed evidence of the impact on both bank-specific and macroeconomic determinants on banks’ profitability. The results of our research show that such bank specific determinants as the bank size and equity to assets have a significant positive effect on Islamic banks’ profitability, while non-performing loans to total gross loans and cost of running operations to operating income have a significant negative effect on both bank types. The results also show that non-performing loans to total gross loans have the greater impact on the profitability of conventional banks. Net loans to total assets do not affect the bank profitability for either Islamic or conventional banks. Taking into consideration macroeconomic determinants, we found that annual GDP growth and annual average oil price have a significant positive effect on Islamic banks’ profitability. GDP growth shows also a positive impact on the profitability of conventional banks. There was no link between the annual consumer price index in the bank’s country and the profitability of the Islamic and conventional banks.

The remainder of this paper is organised as follows: Section 2 presents the background of Islamic banks’ development. Section 3 reviews the existing literature of theories on profit. Section 4 reviews the existing empirical literature on the determinants of bank profitability and hypothesis development. Section 5 presents the data and methodology applied. Section 6 is devoted to results and discussion. Section 7 presents the robustness checks. Section 8 summarises the study and suggests a direction for further studies.

## 2. Islamic banking development

**Fig 1:**
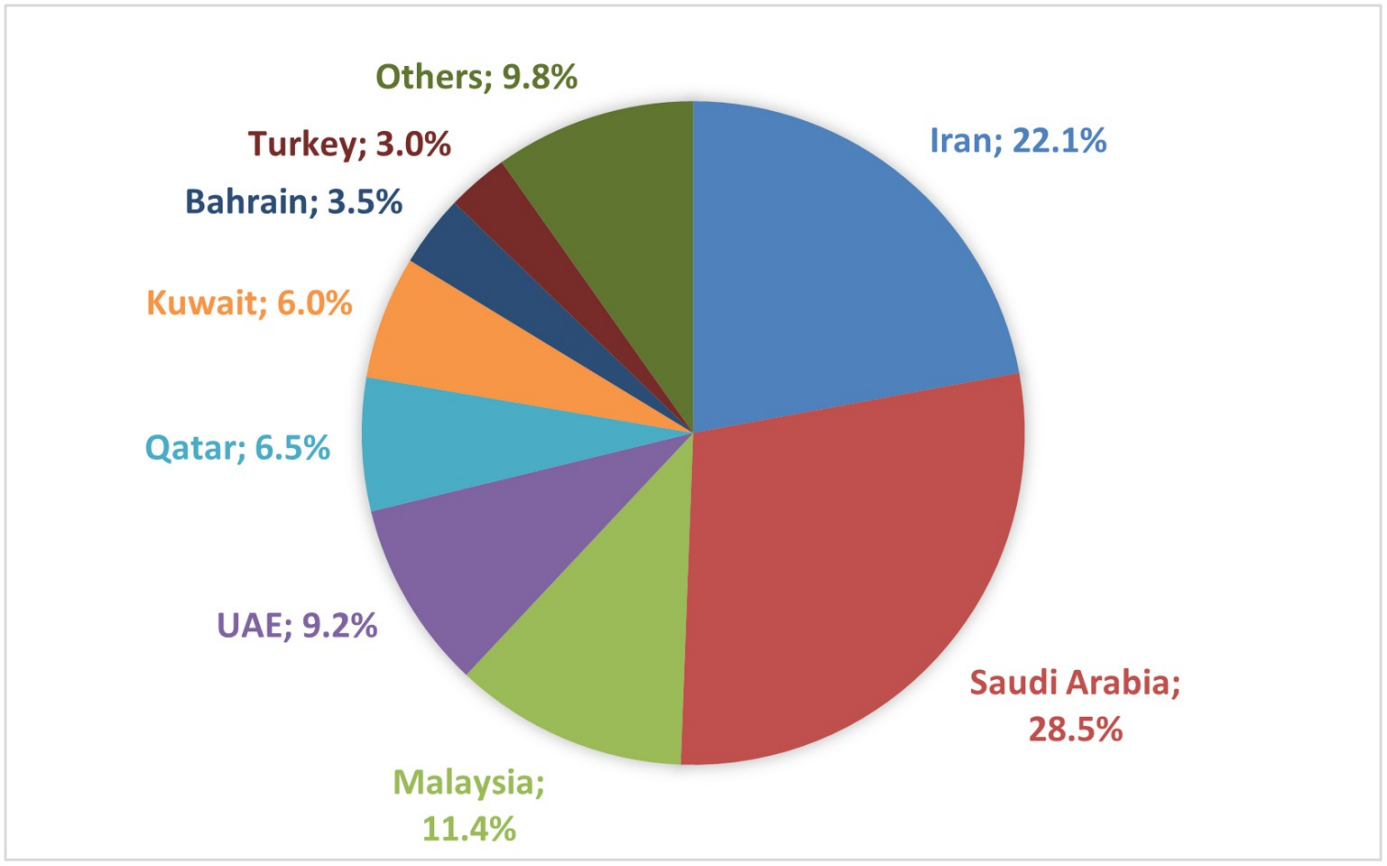
Jurisdiction share of global Islamic banking assets (%) (3Q2020). *Source*: [26].

The significance of Islamic banking has been increasing in the last few decades. Since the 1970s, the assets of Islamic banks have been growing dynamically and at the end of September 2020 reached over US$ 1,8 trillion [26]. The reasons why Islamic banks were established are connected with the neo-revivalist movements among Muslim societies on one hand and the rising wealth of some Middle East countries on the other. One of the most influential movements was the Muslim Brotherhood, established in Al-Ismailijja (Egypt) in 1928 by Hasan al-Banna. The Muslim Brotherhood was critical of the interest-based financial system in Egypt and other parts of the Muslim world and argued that since Islam provides its followers with a comprehensive ideological framework for all aspects of life, economic affairs should also be included within that framework [27]. The opinions of the Muslim Brotherhood and other Muslim movements found followers in the academic world as well as among market practitioners. But if it were not for the wealth of some of the states in the Middle East, which resulted from the oil crisis and the subsequent enormous increases in oil prices, the development of Islamic banking probably would have been much slower. Almost all the Islamic banks that were established in the 1970s were partly or even entirely funded by oil-linked wealth coming from the Middle Eastern countries, mainly Saudi Arabia, the United Arab Emirates, and Kuwait. The establishment of Islamic banks was also influenced by the pan-Islamic movement, which assumed the unification of the Muslim world and which gained significance in the 1970s [28]. Up to now, this region is the leader in the Islamic banking market. In the top 7 countries with the largest Islamic banking assets, 6 are located in the Middle East, with the Kingdom of Arabia and Iran taking 1^st^ and 2^nd^ place, respectively (Fig 1).

An Islamic banking jurisdiction is deemed to be systematically important when the Islamic banking assets in the country account for more than 15% of the assets of its total domestic banking sector or when they hold at least 5% of the global Islamic banking assets [29]. Based on this, eight countries from the Middle East can be considered systematically important Islamic jurisdictions. These are: Iran, Saudi Arabia, Kuwait, Qatar, the United Arab Emirates, Jordan, Bahrain and Oman [26].

The route of development of Islamic banking in the Middle East is slightly different depending on the country. For instance, in the United Arab Emirates, the first legal regulations regarding Islamic finance and banking operations were already set up in the 1980s and fully cover operations and supervision of the institutions, while, for example, in Saudi Arabia there is no direct regulatory framework for Islamic banking [28]. Regardless of the route taken, the Middle Eastern countries have undergone a number of economic, corporate governance, accounting standards, and regulatory reforms, which have created an environment within which Islamic banks can maintain profitable operations [30]. Therefore, it can be assumed that they can be competitive with their conventional counterparts. Thus, it is valuable to examine the determinants which affect the performance of both types of banks, Islamic and conventional, in the Middle East.

## 3. Theories of profit

Profit and profitability are important concepts for accounting scholars, social scientists, practitioners and society in general. They have been examined by the representatives of many sciences including, but not limited to, economic sociology, political economy, sociology, and accounting [31]. Profit is usually understood as the difference between income and expenses. The desire to make profit is a fundamental characteristic of capitalism. The existence, growth, and survival of an enterprise depend upon the profit which it can earn [32]. While profit is an absolute value, profitability in the broad sense of the word means efficiency. It can be measured by such ratios as the return on assets and the return on equity. These two profitability measures reflect how the management uses the organisation’s actual investment resources (assets or equity) to generate profits. Additionally, in banks, the net interest margin ratio is also used to measure profitability. It should be pointed out that indicators of profitability are mandatory in conducting a comparative analysis and evaluation of the financial situation of the enterprise. Additionally, profitability is important in making decisions regarding investments, plans, budgets, coordination, evaluation, and the management of business operations [33].

Regarding the nature of profit in a competitive economy, a number of theories have been put forward over the years. Nearly all of them are fundamentally different from one another and fall short in explaining the true function of profit in a free economy [32]. The majority of these theories consider profit, however, as a reward for some contribution to the value creation process within the organisation [34].

One of the earliest theories of profit was the risk bearing theory formulated by Hawley in 1893 [35], and developed further in later years [36]. In his opinion, the assumption of risk is the distinguishing function of the entrepreneur. And profit is the reward for the risk they assume. Thus, nobody will take a risk if they do not think they will make a profit. There is a positive relationship between risk and profit–the higher the risk, the greater is the possibility of profit; and the smaller the risk, the lower is the possibility of profit. The degree of risk varies across industries. Some of them are riskier than others. According to Hawley, an entrepreneur faces different types of risks, which cannot be insured and thus cannot be avoided. They can result for instance from changes in macroeconomic policy or product obsolescence. If an entrepreneur wants to make a profit, they have to undertake these risks.

Another theory, the dynamic theory of profit, was put forth by Clark [37]. Profit, in his view, is the difference between a price of a product and its production costs. It is the outcome of dynamic change. Thus, a real profit cannot be made in a static economy where risk and uncertainty do not exist and supply and expected demand will always be equal. The reward in a static economy is exactly equal to the marginal productivity of the production elements, hence there is no profit. But as Clark points out, the economy is not static but dynamic. In the dynamic economy, constant changes occur, for example changes in the population’s size, variations in the range of human desires, modifications to industrial processes, etc. These changes impact the demand and supply of products and therefore profit arises.

Another theory of profit was created by Knight [38], who introduced the fundamental element of uncertainty, which allowed the concept of profit to gain importance as an economic concept [31]. According to Knight’s uncertainty bearing theory, profit is the reward for bearing uncertainty. Knight divided the risk into insurable risks and non-insurable risks. The latter is also known as uncertainty. An insurable risk can be forecast, and its probability of occurrence statistically estimated. It is possible to avoid the losses from such risks by paying some premium for an insurance. Thus, the insurable risks do not give rise to uncertainty. The example of such a risk is fire loss. Uninsurable risks, on the other hand, are those occurrences that cannot be forecast or whose likelihood of occurring cannot be easily assessed. As a result, they might cause uncertainty in the production process. Uncertainty is regarded to have an objective nature, be caused by forces beyond the control of any organisation, and have more to do with processes that are playing out at the macro level. No insurance provider is prepared to offer insurance protection against this category of risks. The reward for bearing them is profit.

The innovation theory of profit was proposed by Schumpeter, who argued that anyone seeking profit must innovate. In his view, profit is the reward for successful innovation. The examples of innovations are the launch of a new product, the application of a new method of production, or the sales of a product and opening of a new market [39].

The theories described above are typical for neoclassical economic thought. However, the emphasis on profit and the resulting undesirable effects and behaviour have become a serious concern in the last decades [31]. It has become clear that profits cannot overshadow other aspects of life. Organisations must be conscious how their activities affect the society and environment. Islamic banks are an example of such enterprises, whose goals are beyond making profit. Like other financial institutions, naturally they aim to generate it. However, they should also contribute to the achievement of the socio-economic goals of Islam.

## 4. Empirical literature review and hypotheses development

Determinants of conventional banks profitability, measured by such ratios as return on assets (ROA), return on equity (ROE), or net interest margins (NIM), have been extensively examined since the 1990s. Some of the earliest studies were conducted by Short [40], Bourke [41], Molyneux and Thornton [42], and Berger [43]. The studies of the performance of Islamic banks appeared much later, namely at the end of the first decade of the current millennium, although it should be mentioned that the pioneer of those studies, Sudin Haron, published his first article on Islamic bank profitability already in 1997 [44]. The results of these studies reveal different factors which affect bank profitability. Those factors are usually divided into macroeconomic (external) and bank-specific (internal) factors. Some studies include a third group of determinants, which are called industry-specific factors [19,45,46].

As Al-Homaidi et al. [47] noted, prior studies of a bank’s performance can be classified into three categories. The first category are studies of a bank’s profitability determinants in different countries around the world. For example, such studies were conducted by Demirgüç-Kunt and Huizinga [48], who examined 80 countries, Short [40], who studied 12 countries and Bourke [41], who also studied 12 countries. The second category includes studies that investigate a bank’s performance determinants among different banks in the same region. To this group belong for example studies of Molyneux and Thornton, who focused on Europe [42], Elekdag et al. [49], who studied euro area, Flamini et al. [50], who examined Sub-Saharan Africa and Ben Khediri and Ben Khedhiri [7], Mateev and Bachvarov [51] and Mateev et al. [16] who studied the region of the Middle East and North Africa. Finally, the last and the largest group of studies is focused on the investigation of a bank’s profitability determinants in a single country. Here belong, inter alia, studies of Spain by Trujillo-Ponce [2], Malaysia by Abduh and Idrees [19], Wasiuzzaman and Gunasegavan [21], India by Almaqtari et al. [52,53], Al-Homaidi et al. [47,54], Pakistan by Rahman et al. [55].

Based on the existing literature, this paper attempts to find the macroeconomic and bank-specific determinants which affect Islamic and conventional banks’ profitability, reflected in return on average assets (ROAA) and return on average equity (ROAE) ratios in the region of the Middle East.

### 4.1 Macroeconomic determinants

The macroeconomic determinants are those factors that are beyond the control of a bank but reflect the economic environment that affects the performance of banks. The factors which belong to this group include GDP growth, inflation rate, and oil price.

#### 4.1.1 GDP growth

The GDP growth can be defined as the annual change of the GDP. It is the most general and most direct measure of macroeconomic development [56], which is often used as a variable that reflects the business cycle. Higher GDP growth stimulates demand for bank financing; moreover, in times of better economic conditions, the number of non-repaid loans decreases. Therefore, a positive relationship is expected between GDP growth and the bank’s profitability. Such a relationship was confirmed, inter alia, by Demirgüç-Kunt and Huizinga [48], Bikker and Hu [56], and Flamini et al. [50]. It is also consistent with some studies, which included Islamic banks, for example, studies conducted by Srairi [57] as well as by Wasiuzzaman and Tarmizi [58]. On the other hand, the opposite result, a negative relationship between GDP growth and the bank’s profitabilty was found by Al-Homaidi et al. [54], who investigated performance of conventional banks as well as Mateev and Bachvarov [51], who studied both Islamic and conventional banks. And the lack of a significant correlation between GDP growth and the profitability of Islamic banks was found in the research carried out by Abduh and Idrees [19]. We follow the literature that found a positive relation between GDP growth and the bank’s profitability and, therefore, our first hypothesis is as follows:

*H1*: *GDP growth positively affects the profitability of conventional and Islamic banks*.

#### 4.1.2 Inflation

The relationship between inflation and banks profitability has been profoundly discussed in the literature. Inflation also reflects aspects of the business cycle [59]. It is assumed that inflation has a positive impact on probability since bank income increases more with inflation than costs. In an inflationary environment, banks also receive higher earnings from delays in crediting customer accounts [18]. The positive influence of the inflation variable on bank performance was confirmed by Bourke [41], Molyneux and Thornton [42], and Demirgüç-Kunt and Huizinga [48]. Some studies on Islamic banks showed the same results [20,58]. It should be pointed out, though, that this happens when inflation is anticipated [50]; however, if banks fail to capture inflation forecasts and adjust profitability accordingly [23] then there is a possibility that bank costs may increase faster than revenues and, as a result, negatively affect bank performance [18]. Such a negative relationship between inflation and profitability was found in some studies on Islamic banking, including the studies conducted by Muda et al. [60] and Zeitun [61]. According to Eljelly [18], such results can be explained by the fact that in the case of Islamic banks, the process of inflation anticipation is difficult since the profit-sharing mechanism exposes banks to a share of this inflation effect. Conventional banks can adjust interest rates accordingly, hence revenues have a better chance to increase more than costs.

Accordingly, our second hypothesis is as follows:

*H2*: *There is a positive relationship between the inflation rate and banks profitability for conventional banks and a negative relationship for Islamic banks*.

#### 4.1.3 Oil price

Oil is a significant source of revenue for Middle Eastern countries and the main reason for the emergence and growth of Islamic banking in this region of the world. However, the oil price is not a variable that is usually taken into account in the studies of bank performance. A study that examined the relationship between oil price and bank profitability in the Gulf Cooperation Council (GCC) region was conducted by Esmaeil et al. [62]. Their analysis showed that oil prices significantly affect the profitability of both conventional banks and Islamic banks, and this was consistent with the outcomes of research conducted by Ali et. al. [20]. An extensive study examining the relationship between oil price shocks and bank profitability in the Middle East and North Africa (MENA) region was also conducted by Poghosyan and Hesse [63]. They distinguished two channels through which oil price shocks may affect bank profitability, namely, a direct and indirect channel. In the direct channel, oil price shocks could affect bank profitability directly via increased oil-related lending or business activity. Indirectly, they are transmitted through the macroeconomic and institutional characteristics of countries supported by increased expectations and business sentiment in those countries. The authors found that in the MENA region, oil price shocks affect profitability through the indirect channel. Their study also showed that oil price shocks have an impact mainly on investment banks, which can be explained by the fact that the price largely affects investment activity in the examined region.

Since the growth of Islamic banks has been funded by export of oil, our third hypothesis is as follows:

*H3*: *Oil price increase positively affects only the profitability of Islamic banks*.

### 4.2 Bank-specific factors

Bank-specific factors are determinants that are predominantly influenced by the decisions of a bank’s management and its policy objectives. Such factors are the size of the bank, level of liquidity, capital adequacy, asset quality, and efficiency.

#### 4.2.1 Size of the bank

Bank size is measured in terms of the total assets of the bank. Empirical studies on the influence of bank size show inconclusive results. On the one hand, the higher the value of a bank’s assets, the lower its operating costs due to the economies of scale; therefore, higher profitability can be expected as the bank’s assets increase. On the other hand, extremely large banks may achieve lower profitability, e.g., due to bureaucratic procedures. As far as conventional banks are concerned, the positive and significant influence of bank size on its profitability has been demonstrated, inter alia, by Demirgüç-Kunt and Huizinga [48], Khrawish [64] as well as Flamini et al. [50]. However, the opposite result, a negative and significant relationship between the bank’s size and its profitability, was found by Aladwan [65] and Al-Homaidi et al. [47]. On the other hand, Kolapo et al. [66] and Anarfi et al. [67] found that the size of the bank has no significant impact on its profitability. As far as Islamic banks are concerned, a positive and significant influence of bank size on its profitability was found by Abduh and Idrees [19], Srairi [57] as well as Al-Qudah and Jaradat [68], while studies conducted by Wasiuzzaman and Tarmizi [58] showed no significant relationship between the size of the bank and profitability.

We follow the literature that found a positive relation between bank size and the bank’s profitability and, therefore, our fourth hypothesis is as follows:

*H4*: *Bank size positively affects the profitability of conventional and Islamic banks*.

#### 4.2.2 Liquidity

Bank liquidity can be measured by net loans to total assets ratio, a factor that is expected to have a positive impact on bank performance. Financing provided by banks is the main source of a bank’s income, therefore, the higher its level, the higher the bank’s profitability. But, on the other hand, a high level of this ratio may contribute to a decrease in the bank’s liquidity and an increase in the number of outstanding loans. In such a situation, the bank’s profitability may decline. A positive and significant impact of the net loan to assets ratio was found in the studies by Trujillo-Ponce [2] as well as the studies by Fah and Hassani [69], which included not only conventional but also Islamic banks. This result was also confirmed by Wasiuzzaman and Tarmizi [58] and Zarrouk et al. [25], who concentrated solely on the study of Islamic banks.

Accordingly, our fifth hypothesis is as follows:

*H5*: *Bank liquidity positively affects the profitability of conventional and Islamic banks*.

#### 4.2.3 Capital adequacy

Capital adequacy is usually measured by the equity to assets ratio, which can be interpreted as the bank’s capital strength or insolvency risk [7]. A high level of this ratio increases the security of both the bank itself and its customers (depositors). In addition, equity is a source for covering any losses incurred by the bank. Moreover, the greater the share of equity, the lower the bank’s need for external capital. On the other hand, an excessively high level of equity may reduce the bank’s ability to increase its profitability. A bank is considered to be more efficient the lower the level of equity capital that is required—while ensuring an adequate level of liquidity and solvency. Research conducted by Berger [43] shows a positive and significant impact on the profitability of the equity to assets ratio. Such a result was also found by Flamini et al. [50] as well as Zarrouk et al. [25], who also examined Islamic banks. On the other hand, the research conducted by Wasiuzzaman and Tarmizi [58] indicates the existence of a negative and significant relationship between the equity to total assets ratio and the profitability of Islamic banks.

We follow the literature that found a positive relation between capital adequacy and the bank’s profitability and, therefore, our sixth hypothesis is as follows:

*H6*: *Capital adequacy positively affects the profitability of conventional and Islamic banks*.

#### 4.2.4 Asset quality

Another variable that should be included in the study of the determinants of bank profitability is the asset quality ratio, which can be defined as an estimation of the quality of a bank’s assets and is normally based on the loans and leases. The asset quality ratio is used in evaluating the credit risk associated with assets. Generally, the lower the ratio, the better the asset quality of the bank [33]. As proxies of asset quality, ratios such as loan loss reserve to gross loans or non-performing loans to gross loans are used. According to Heffernan and Fu [70], the influence of the loan loss reserve to gross loans ratio on bank performance is ambiguous and not necessarily negative; on the one hand, higher provisioning signals the likelihood of possible losses of loans in the future, while on the other, it can also indicate a timely recognition of weak loans by banks. And it is the latter that is indicated in the studies of Heffernan and Fu. The opposite and expected outcome, a negative and significant relationship between the loan loss reserve to gross loans ratio and bank profitability, was found by Wasiuzzaman and Tarmizi [58]. On the other hand, Youseff and Samir, who also used the same proxy as asset quality, found no significant relationship between this ratio and conventional and Islamic bank profitability [71]. Expected results were seen in studies that used non-performing loans to gross loans as a measure of asset quality, for example, in the research conducted by Ally [72] and by Detragiache et al. [73]. Both studies found that the impact of the non-performing loans to gross loans ratio (examined variable) on bank profitability is both significant and negative.

Accordingly, our seventh hypothesis is as follows:

*H7*: *There is a negative relationship between share of non-performing loans in the total gross loans and the profitability of conventional and Islamic banks*.

#### 4.2.5 Efficiency

Efficiency ratios are used to analyse how well the bank is managing and controlling its assets [25]. Standard measures of efficiency include cost to income and cost to assets ratios, sporadically differentiating between personnel and non-personnel costs [42]. Most studies of both conventional and Islamic banks show that the lower the ratio (the better efficiency), the greater the profitability of the bank [7,23,49,70,74]. However, the opposite results were demonstrated by Molyneux and Thornton [42], who used staff expenses as a percentage of the total assets ratio as a proxy of efficiency. They explained that this counter-intuitive outcome suggested that high profits earned by banks in a regulated industry may be appropriated in the form of higher payroll expenses [42]. Molyneux and Thornton’s results were confirmed by Eljelly [18], who studied the determinants of Islamic bank profitability using a cost to income ratio as a proxy of efficiency and concluded the findings as rather easy to explain. Namely, the increased cost of investments in human resources development brings greater profitability in both the medium- and longer-terms.

We follow the literature that found a negative relationship between the cost of running operations divided by operating income and the bank’s profitability and, therefore, our eighth hypothesis is as follows:

*H8*: *There is a negative relationship between the cost of running operations divided by operating income and the profitability of conventional and Islamic banks*

## 5. Method

**Fig 2:**
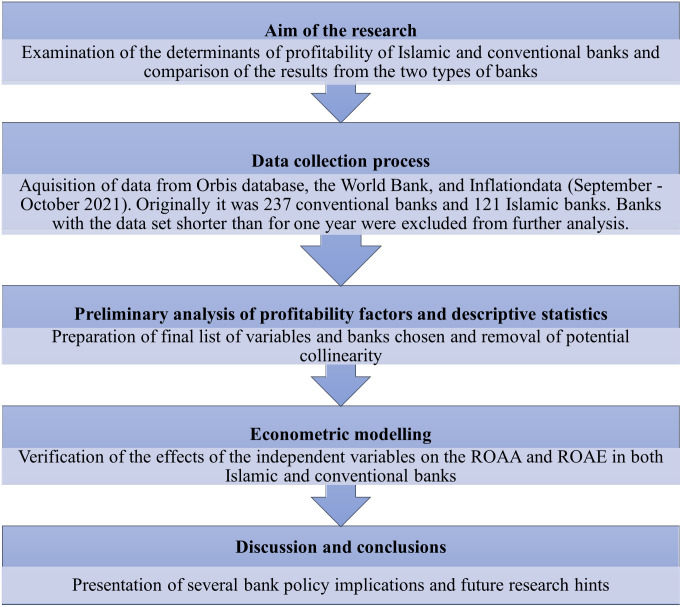
The research path. Source: Self-study.

In the first stage, a goal was formulated to which the scope of the study was subordinated. Due to the need to obtain detailed information, it was decided to use the Orbis database, which allowed for high-quality data to be obtained from banks financial statements. Factor analysis was then carried out after processing the collected data, and applied to rule out potential collinearity and reduce dimensions, bearing in mind we started with 358 banks from 12 countries, 18 explanatory variables, and 2 dependent variables–ROAA and ROAE. As a next step, descriptive statistics were calculated and panel models were constructed to verify the effect of the independent variables on the ROAA and ROAE for both Islamic and conventional banks (Fig 2).

Our research was initiated by the discovery of a gap in the literature focusing on the differences between Islamic and conventional banks, especially related to the ambiguous determinants of profitability amongst Middle East banks. We noticed that many studies have not come to the same conclusion because surveys were conducted in different countries, in different periods, and taking into consideration different econometric methods. Differences in the business models between conventional and Islamic banks can lead not only to profitability differences, but also to cost-effectiveness or capital stability differences. We based our study on the experience and limitations of previous studies performed by the researchers in the past [75].

It is worth emphasising that despite the fact that some researchers have proved the existence of certain differences between Islamic and conventional banks, the differences they have indicated between the two types of banking do not translate into their financial states and generally the same profitability factors could be observed being measured by ROAA and ROAE. Based on the research carried out by Farah [76], it may also be caused by accounting standards that may not be able to spot differences in the business models. Moreover, the financial statements of both sectors show similar exposures, dominated by items related to the financing of government and significant commercial projects, holding of treasury securities, bonds, etc.

### 5.1 Data collection process

The bank-level data of the Islamic and conventional banks’ accounting statements were retrieved from Orbis. The dataset covers 12 countries located in the Middle East, namely Bahrain, Iran, Iraq, Jordan, Kuwait, Lebanon, Oman, Qatar, Saudi Arabia, the Syrian Arab Republic, the United Arab Emirates, and Yemen. The total value of Islamic bank assets in these countries in the sample is US$ 1,344,009,577 in 2019 (versus 2,282,198,992 for conventional banks). The market is characterised by a relatively high-income level and competition that is intensifying with a high percentage of customers whose primary concern is product purity. The region has an overall high level of economic development, but its countries range from being very poor, such as Yemen, to being extremely wealthy, such as Qatar, the United Arab Emirates, and Kuwait. It is highly reliant on oil and four of the countries were among the top ten oil exporters in 2020; Saudi Arabia, Iran, the United Arab Emirates, and Kuwait. Jordan and Lebanon are the only net oil importers among the region in the sample. The largest oil reserves are to be found in Saudi Arabia, with 20 per cent of the world’s known oil reserves [77]. The macro-level data were extracted from the World Bank and the oil price from Inflationdata. The sample covers the period 2012–2020 and a total of 270 banks over nine years, with at least one year with full observations for each explanatory variable. We began with 358 observations (121 Islamic banks and 237 conventional banks) of the dependent variables, ROAE and ROAA, but due to missing values and errors in the data, the final number of observations is 270. Banks with the data set shorter than for one financial year were excluded from further analysis (Table 1).

**Table 1:** Number of Islamic and conventional banks used in the analysis over the period 2012–2020.

	Islamic Bank—raw data	Conventional Bank—raw data	Islamic Bank—final data	Conventional Bank—final data
**Bahrain**	15	16	15	10
**Iran, Islamic Rep.**	33	3	30	3
**Iraq**	17	28	17	24
**Jordan**	5	18	5	13
**Kuwait**	11	7	11	6
**Lebanon**	3	70	2	36
**Oman**	3	12	3	7
**Qatar**	7	10	5	7
**Saudi Arabia**	9	13	6	11
**Syrian Arab Republic**	3	13	3	12
**United Arab Emirates**	10	36	10	25
**Yemen, Rep.**	5	11	4	5
**Total**	**121**	**237**	**111**	**159**

Source: Self-study based on Orbis.

**Fig 3:**
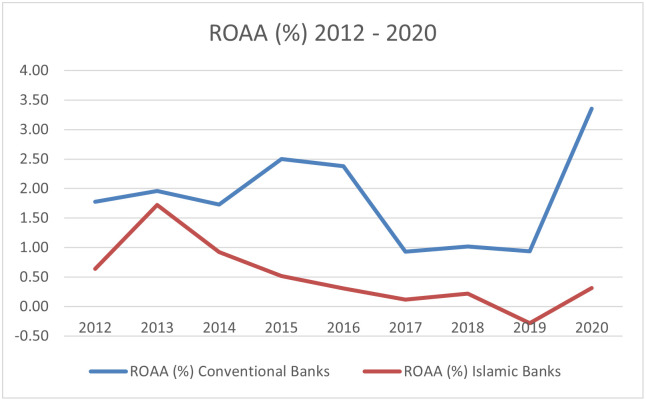
Average ROAA among Islamic banks and conventional banks 2012–2020. Source: Self-study based on Orbis.

**Fig 4:**
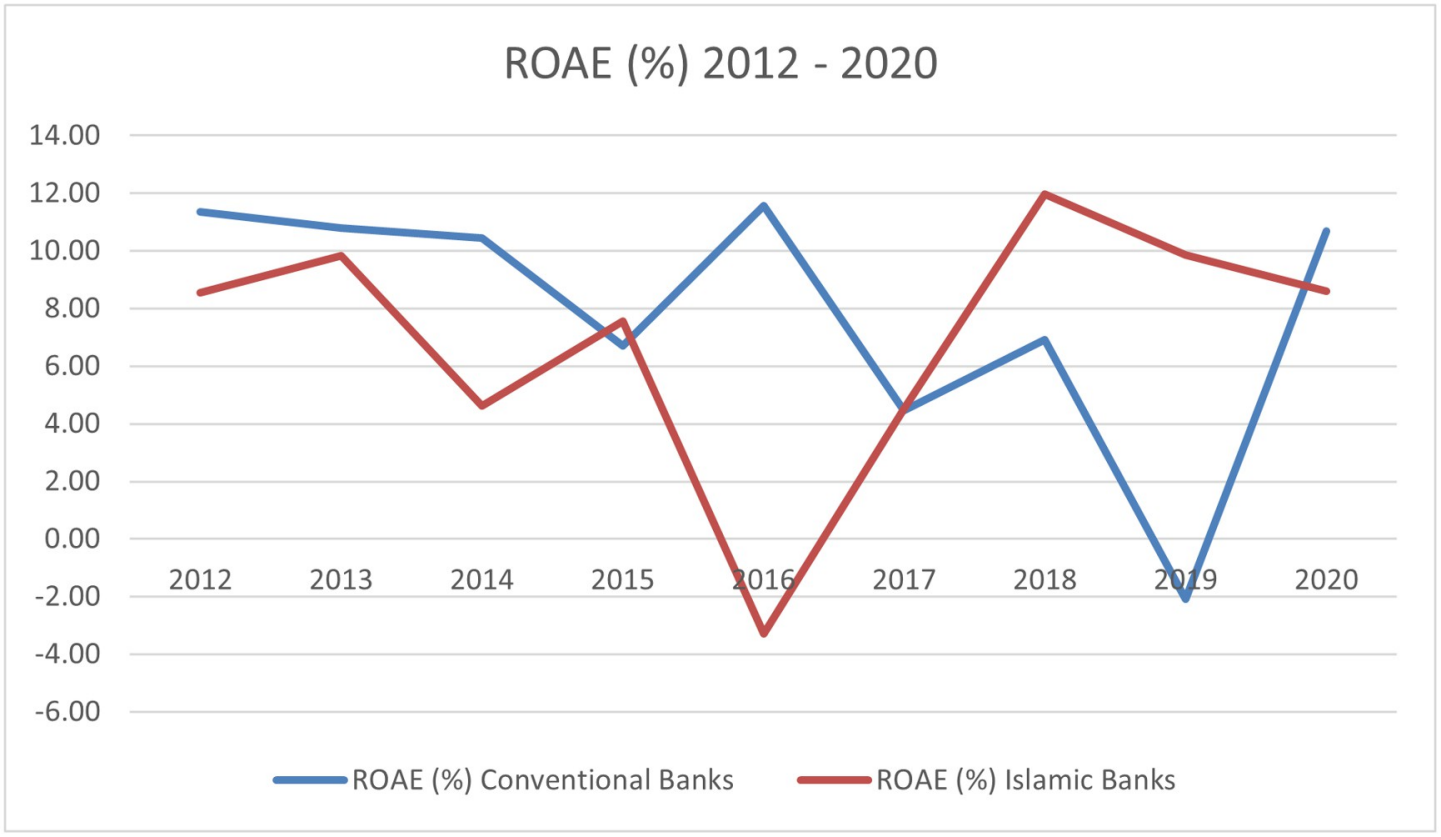
Average ROAE among Islamic banks and conventional banks 2012–2020. Source: Self-study based on Orbis.

The empirical literature on bank performance focuses primarily on assessing the relationship between bank performance measures such as ROE and ROA and several predictors of bank performance based on fundamental data such as size of bank, share of non-performing loans, level of operational costs, etc., as well as general macroeconomic factors such as GDP growth, inflation, interest rates. In order to measure the profitability of banks, we use two measures that are commonly used in the literature, namely return on average assets (ROAA) and return on average equity (ROAE). While ROAA is measured as net income to total assets, ROAE is measured as net income to shareholders’ equity. These two profitability measures reflect how banking management uses the bank’s actual investment resources (assets or equity) to generate profits. The evolution of these profitability measures is shown in Figs 3 and 4.

ROAA is determined by dividing net profit after taxes by total assets. This measurement of the company’s operating efficiency is based on the profits generated by the company from its total assets. The ratio can be calculated by applying the following formula: Net profit after taxes/average assets.

Based on our sample, conventional banks are more profitable than Islamic banks; through the entire period, ROAA for conventional banks was higher than for Islamic banks. This could be driven by high assets and larger market size [78].

It is worth noting that only for Islamic banks was ROAA negative in 2019 (Table 2). In the analysed period, ROAA for conventional banks increased from 1.78% in 2012 to 3.35% in 2020. We can observe the reverse situation for Islamic banks, where ROAA decreased from 0.64% in 2012 to 0.31% in 2020. Islamic banks appear to have performed worse than conventional banks when it comes to the ROAA profitability measure. Surprisingly, our study shows that the first year of the COVID-19 pandemic had no significant effect on bank profitability. We have observed that both average ROAA and ROAE saw a decline in 2019 but increased in 2020. Only ROAE for Islamic banks slightly decreased in 2020.

**Table 2:** ROAA (%) comparison among Islamic banks and conventional banks 2012–2020.

Year	Islamic banks				Conventional banks			
	Min	Max	Mean	SD	Min	Max	Mean	SD
2020	-24.34	11.33	0.31	4.97	-6.30	69.48	3.35	10.37
2019	-32.00	7.06	-0.28	4.75	-5.78	24.63	0.94	2.88
2018	-16.29	42.58	0.22	5.71	-9.51	23.45	1.02	2.55
2017	-18.97	28.49	0.12	4.41	-12.01	21.01	0.93	3.00
2016	-13.31	13.76	0.31	3.55	-3.13	31.43	2.38	4.10
2015	-31.14	6.48	0.52	4.60	-7.39	34.09	2.50	4.47
2014	-7.12	5.71	0.92	2.32	-14.57	19.96	1.73	2.63
2013	-9.93	22.08	1.72	3.47	-5.29	29.39	1.96	3.01
2012	-38.92	7.31	0.64	5.83	-2.07	8.03	1.78	1.61

Source: Self-study based on Orbis.

ROAE is calculated by dividing net profit after tax by total shareholder equity. This ratio determines the rate of return on a company’s investment for its shareholders. To calculate this indicator, the following formula is applied [79]: Net profit after taxes/ Average total shareholders’ equity.

From 2012 to 2015, ROAE for both Islamic and conventional banks was similar, on a positive level. It can be seen that in 2016, ROAE for Islamic banks was negative. A negative ROAE is clearly interpreted as an unfavourable financial situation for the company, but in the case of Islamic banks it must be remembered that some of the banks are relatively new, so they could show losses and at the same time be supplied with capital from the investor-founder. This is evidenced by the fact that since 2017, a continuous increase in the indicator can be observed, and only in 2016 was negative ROAE seen within the analysed period. A similar situation was observed for conventional banks, where, in 2019, negative ROAE was observed (Table 3).

**Table 3:** ROAE (%) comparison among Islamic banks and conventional banks 2012–2020.

Year	Islamic banks				Conventional banks			
	Min	Max	Mean	SD	Min	Max	Mean	SD
2020	-208.57	91.59	8.61	40.99	-52.71	144.52	10.69	33.48
2019	-90.83	217.49	9.87	30.14	-674.56	31.29	-2.09	60.67
2018	-126.64	264.41	11.97	43.50	-122.74	219.11	6.92	23.78
2017	-447.97	216.62	4.52	58.32	-143.98	31.63	4.45	16.86
2016	-840.84	132.68	-3.30	87.65	-7.57	63.83	11.56	12.29
2015	-39.68	51.55	7.56	12.79	-827.06	77.22	6.72	72.44
2014	-106.01	28.64	4.62	19.17	-49.60	42.21	10.44	10.29
2013	-30.13	47.03	9.82	13.59	-18.28	41.59	10.80	8.24
2012	-42.31	47.78	8.54	15.37	-12.95	47.80	11.34	7.88

Source: Self-study based on Orbis.

To summarise, ROAE is affected by ROAA as well as by the bank’s degree of financial leverage. As returns on assets, in general, tend to be lower for financial institutions, most banks often use financial leverage to increase the return on equity to a competitive level and this is a trend we have observed for both the Islamic and the conventional banks.

It would seem that the situation should be less complex as soon as Islamic banks are engaged in banking and financial activities in the country, because the Islamic bank then applies sharia rules. However, in a financial system where we see the coexistence of conventional and Islamic banks, the task is not so simple for an Islamic bank, which has to compete with the conventional banks. Therefore, even if we compare two Islamic banks with the same activities, selling the same products and following the same rules, but operating in two different countries, their financial results and operational goals may differ significantly [80]. This is one of the reasons why we have observed in our sample that the standard deviation for Islamic banks was significantly higher than for conventional banks for both ROAA and ROAE.

### 5.2 Model and variables

As already mentioned above, our research sample ultimately consisted of 111 Islamic banks and 159 conventional banks. For both groups, the *Orbis* database enabled the acquisition of data for the period 2012–2020, however, the obtained panel dataset was unbalanced. This meant that for some banks, data on selected ratios or selected years were unavailable. Missing data caused some cases to be removed from the dataset during the final model estimation.

In our study, we included two measures of bank financial performance which we used as dependent variables in the model (see Table 6). The first is the ROAA ratio, and the second is the ROAE ratio.

Based on the literature review, we included a set of independent variables in the model, which we have divided into bank-specific factors and macroeconomic factors [81]. The definitions of the variables and the expected sign of the coefficient in the models are presented in Table 4. We also provide a rationale behind the usage of the variables in the following part of this section.

**Table 4:** Definitions of the research variables.

Variable	Definition	Expected sign of the coefficient for Islamic banks	Expected sign of the coefficient for conventional banks
*ROAA*	Profit or loss (net) divided by average annual assets (%).	n/a	n/a
*ROAE*	Profit or Loss (net) divided by average annual equity (%).	n/a	n/a
*LogTA*	Decimal logarithm of total assets.	+	+
*ETA*	Equity divided by total assets (%).	+	+
*NLTA*	Net loans divided by total assets (%).	+	+
*CI*	Cost of running operations divided by operating income (%).	-	-
*NPL*	Non-performing loans divided by total gross loans (%).	-	-
*GDP*	Change in GDP in the bank’s country compared to the previous year (%).	+	+
*CPI*	Annual consumer price index in the bank’s country (%).	-	+
*OP*	Annual average oil price (USD/barrel).	+	+/-

The **size of a bank** (variable *LogTA*) is usually measured as the logarithm of total assets. The natural logarithm of total assets is taken as a proxy of bank size, which is frequently used in empirical literature [43,45,74]. However, there is a contradiction among researchers about the behaviour of size towards profitability. Large banks, with a high asset value, typically reduce costs by taking advantage of economies of scale and scope, which can help improve profitability. On the other hand, a bank’s profit can only increase to a certain limit and then it starts to decline. Often the reason for this decline is excessive bureaucracy.

**Equity divided by total assets** (variable *ETA*) represents the group of capital structure indicators. It should be noted that a high share of equity in the bank’s assets increases the security of both the bank itself and its customers who have entrusted the bank with their money. In addition, equity is a source of coverage for potential losses. The greater the share of equity, the lower the bank’s need for external capital. However, too high a level of equity capital may reduce the bank’s ability to increase its profitability. Dermirgüç-Kunt and Huizinga [48], in their studies of the determinants of bank profitability, found a statistically significant positive relationship between equity to total assets and profitability. On the other hand, Hassan and Bashir [82] found evidence of a negative relationship between the equity ratio and return on equity. Taking into consideration our study, we expect to see positive affect of *ETA* on conventional and Islamic banks profitability.

The next indicator, **net loans divided by total assets** (variable *NLTA*), should have a positive effect on profitability for both types of banks. Bank loans are the main source of a bank’s income, therefore, the higher the level of financing provided by banks, the higher the bank’s profitability should be.

**Cost of running operations divided by operating income** (variable *CI*) is expected to be negatively related to profitability of both conventional and Islamic banks, as it increases the expenses of each type of the bank. Efficient banks should be able to reduce their operating costs successfully which ultimately increases their profitability.

**Non-performing loans divided by total gross loans** (variable *NPL*) is expected to be negatively correlated with the profitability of both conventional and Islamic banks. Usually, the loan is perceived as non-performing when the borrowers fail to return principal and/or interest within a specified period, which is normally 3 months or 90 days. Scholars also denote non-performing loans as problematic or bad loans. In this study, the ratio of non-performing loans to total gross loans has been used [83].

**Change in GDP in the bank’s country compared to the previous year** (variable *GDP*) is used as a proxy of the economic cycle. It is expected that the profitability of both Islamic and conventional banks will rise in good economic times and decrease in recessions. These effects are expected as credit quality will decrease in recessions and, therefore, as the risk of default will rise, the bank returns are expected to be lowered. In addition, higher rates of GDP growth can stimulate higher demand for financing offered by banks.

**Annual consumer price index in the bank’s country** (variable *CPI*) is expected to positively affect profitability of conventional banks. Inflation affects banks pricing behaviour, and hence, if banks expect general inflation to be higher in the future, they may believe that they can increase their prices without experiencing a decline in demand for their output. However, it is also expected see the negative link between annual consumer price index in the bank’s country and profitability of Islamic banks.

Concerning **annual average oil price** (variable *OP*), a positive impact on Islamic banks profitability is expected as the Middle East countries are mainly oil exporters.

In order to investigate the relationship between dependent and independent variables, we used both static and dynamic panel modelling. The static modelling of panel data makes it possible to distinguish individual effects from effects caused by external factors. It becomes possible to control the influence of the individual, internal differentiation of entities. In the case of panel data, fixed effects (FE) and random effects (RE) models are most often used. Both have their strengths and weaknesses. FE models constitute an appropriate specification if the research process is focused on a selected group of entities. They are most often used for the so-called long panels with a small number of entities and a long test period. RE models are appropriate if the panel is a sample from which conclusions are drawn for the entire population. RE models assume that the error term can vary randomly [84]. In our case, the static approach assumes that there is no persistence in the financial performance of banks. Until now, it has been used in modelling the financial performance of both Islamic [82] and conventional banks [59]. We applied the following form of the static model:

$$Y_{i,t}=\beta_{0}+\sum_{k=1}^{n}\beta_{k}X_{k,it}+\sum_{l=1}^{m}\beta_{l}X_{l,it}+\alpha_{i}+\varepsilon_{i,t},$$ (1)

where:

- *Y*_*i*,*t*_ represents the dependent variable for the bank *i* in the time *t*,
- *β*_0_ represents the constant term,
- *X*_*k*,*it*_ is the *k*-th independent bank-specific variable for the bank *i* in the period *t*,
- *X*_*l*,*it*_ is the *l*-th independent macroeconomic variable in the country of the bank *i* in the period *t*,
- *β*_k_ represents the parameter for *k*-th independent bank-specific variable,
- *β*_*l*_ represents the parameter for *l*-th independent macroeconomic variable,
- *α*_*i*_ is an individual-specific effect for the bank *i* that does not vary across time,
- *ε*_*i*,*t*_ is the error term which assumes different values for each bank at each point in time.

In turn, dynamic panel models assume that there is a persistence of banks’ financial performance. The persistence of bank profits reflects impediments to market competition, informational inefficiency, and/or sensitivity to macroeconomic shocks [85]. The dynamic modelling approach is also widely used in the literature to study the financial performance of Islamic [25,81,86] and conventional banks [25,45]. We applied the following form of the dynamic model:

$$Y_{i,t}=\beta_{0}+{\delta Y}_{i,t-1}+\sum_{k=1}^{n}\beta_{k}X_{k,it}+\sum_{l=1}^{m}\beta_{l}X_{l,it}+\alpha_{i}+\varepsilon_{i,t},$$ (2)

where:

- *Y*_*i*,*t*-1_ represents the one-period lagged dependent variable for the bank *I*,
- *δ* represents the speed of adjustment to equilibrium.

A statistically significant value of the parameter *δ* means that there is a persistence of financial performance. A value close to 0 means that the industry is fairly competitive (a high speed of adjustment), while a value of *δ* close to 1 implies a less competitive structure (very slow adjustment) [45]. In this study, we chose both the static and dynamic approach because we did not want to assume a priori the presence or absence of persistence in bank financial performance.

To assess the validity of estimating separate models for Islamic and conventional banks, we conducted the Chow test [87] for the compared coefficients on both dependent variables. The test statistic value F(9, 1213) for ROAA was 20.96, indicating that conducting separate estimations for both types of banks significantly improves the model fit. The same conclusions were drawn for the variable ROAE, which had a test statistic value of F(9, 1212) equal to 7.14.

## 6. Results and discussion

In this section, we analyse bank-specific and macroeconomic factors affecting the ROAA and ROAE of Islamic and conventional banks. Descriptive statistics for all variables are presented in Table 5. The final database was unbalanced. The variable with the least observations in the created database was NPL. Islamic banks had on average lower ROAA values than conventional banks. For Islamic banks, the standard deviation of ROAA values was also higher. Similarly, in the case of ROAE, Islamic banks showed a lower average value and a higher standard deviation.

**Table 5:** Descriptive statistics.

Variable	Panel A: Islamic banks				
	Obs.	Mean	Std. Dev.	Min	Max
*ROAA*	832	0.359	5.049	-51.420	42.577
*ROAE*	831	6.868	44.051	-840.844	264.412
*LogTA*	832	6.410	0.900	3.624	8.262
*ETA*	832	26.116	30.739	-216.497	99.970
*NLTA*	798	48.544	23.997	-6.347	97.378
*CI*	799	77.688	85.328	-479.250	948.575
*NPL*	513	13.799	36.789	0.000	538.436
*GDP*	939	1.564	5.577	-27.994	13.936
*CPI*	892	7.203	11.259	-3.749	84.864
*OP*	998	58.351	21.924	32.250	91.170
Variable	Panel B: conventional banks				
	Obs.	Mean	Std. Dev.	Min	Max
*ROAA*	1,178	1.810	4.436	-14.569	69.477
*ROAE*	1,177	7.725	36.047	-827.064	219.106
*LogTA*	1,189	6.443	0.903	3.299	8.449
*ETA*	1,189	23.095	21.874	-1.136	99.957
*NLTA*	1,135	41.578	21.792	0.000	97.624
*CI*	1,123	52.357	47.248	-97.180	819.307
*NPL*	964	15.231	24.465	0.016	231.617
*GDP*	1,284	1.249	5.887	-27.994	13.936
*CPI*	1,281	5.020	14.690	-3.749	84.864
*OP*	1,440	58.380	21.927	32.250	91.170

To begin with, we performed a correlation and multicollinearity analysis (see Supporting information, S1 and S2 Tables). The obtained results showed that the problem of multicollinearity of independent variables does not occur.

We initially estimated static and dynamic models for both Islamic and conventional banks. We used the two-step system generalised method of moments estimator in the dynamic approach [88]. In this analysis lagged values of the endogenous dependent variable were treated as instruments in the first stage of estimation and the residuals were treated as instruments in the second stage of estimation. However, in the case of Islamic banks, the parameter for the lagged ROAA is not statistically significant. Moreover, the results of the Arellano-Bond test show that there is no autocorrelation in residuals of the first order. Additionally, the estimated dynamic models for ROAE did not meet the required assumptions. The Sargan test for over-identifying restrictions in dynamic model estimation does reject the null hypothesis that the over-identifying restrictions are valid. Only in the case of conventional banks for ROAA does the dynamic model meet the required assumptions (see Supporting information, S3 Table). For conventional banks, the parameter for the lagged ROAA variable is statistically significant at the 1% level. The results of the Arellano-Bond test show the presence of autocorrelation in residuals of the first order but there is no problem with the autocorrelation in residuals of the second order. Additionally, the Sargan test for over-identifying restrictions in dynamic model estimation does not reject the null hypothesis that the over-identifying restrictions are valid. The above observations suggest that in the case of conventional banks there is a much stronger ROAA persistence. It follows that the Islamic banking industry has a more competitive structure. Conversely, the conventional banking industry may show a less competitive structure and a higher susceptibility to macroeconomic shocks.

However, due to the fact that most of the estimated dynamic models did not meet the assumptions, we based our further analysis on the static approach. The Hausman test shows that the FE model is suitable for both Islamic and conventional banks in this case (χ^2^(8)>33.79 in the case of ROAA models, χ2(8)>18.26 in the case of ROAE models). Due to the presence of heteroscedasticity, we used robust standard errors in the estimation. Table 6 presents the results of the estimation for ROAA as the dependent variable. It can be seen that there are differences in the impact of bank-specific factors on the ROAA of Islamic and conventional banks. The FE model shows that the ROAA of Islamic banks is positively related to bank size (parameter statistically significant at the level of 1%). This relationship did not occur for conventional banks. Our findings offer partial empirical support for H4, which suggests that the bank size affects profitability, but only in the case of the Islamic banks.

**Table 6:** Determinants of profitability measured by ROAA.

Dependent variable: return on average assets (*ROAA*)		
Explanatory variables	Islamic banks	Conventional banks
*LogTA*	4.747*** (1.748)	-0.555 (1.134)
*ETA*	0.132*** (0.041)	0.047 (0.036)
*NLTA*	0.013 (0.022)	-0.006 (0.012)
*CI*	-0.032*** (0.012)	-0.030*** (0.006)
*NPL*	-0.044* (0.026)	-0.047*** (0.017)
*GDP*	0.068** (0.031)	0.048*** (0.016)
*CPI*	0.017 (0.018)	0.007 (0.006)
*OP*	0.013*** (0.004)	0.003 (0.003)
*Constant*	-32.534** (12.721)	6.294 (8.749)
Observations	440	791
Number of banks	76	117
Within R^2^	0.43	0.42
Between R^2^	0.05	0.20
Overall R^2^	0.18	0.30
Hausman-test	χ^2^(8) = 186.23 *p*-value = 0.00	χ^2^(8) = 33.79 *p*-value = 0.00

Note: This table presents the results of the fixed effect models for Islamic and conventional banks (dependent variable ROAA).

***, **, and * indicate statistical significance at the 1%, 5%, and 10% levels, respectively. Robust standard errors are presented in parentheses.

We also observed a difference in the impact of bank capital adequacy on ROAA. Higher capital adequacy (*ETA*) positively influenced Islamic banks and did not have an impact on conventional banks. Our findings offer partial empirical support for H6, which suggests that capital adequacy positively affects profitability but only in the case of Islamic banks.

In the case of both Islamic and conventional banks, we did not observe any impact of the bank’s liquidity on ROAA. Thus, we reject H5.

However, we did observe a strong negative impact of the cost to income (*CI*) ratio on both Islamic and conventional banks. Hence, we accept H8.

We believe there is also an impact of asset quality on ROAA for both Islamic and conventional banks. The FE models confirm the existence of a negative relationship between the *NPL* variable and ROAA. It seems, however, that in the case of Islamic banks, the impact of asset quality on financial efficiency may be slightly weaker but our empirical research allows us to accept H7.

There are some similarities in the impact of macroeconomic variables on the financial performance of Islamic and conventional banks; the impact of GDP growth is positive for both Islamic banks and conventional banks. Thus, we accept H1.

We did not observe any impact of inflation on the financial performance of banks. Thus, we reject H2.

However, we did observe a positive relationship between oil prices and the ROAA of Islamic banks. The parameter for the *OP* variable is statistically significant at the level of 1%. For conventional banks, the parameter for this variable is not statistically significant at any of the standard significance levels. Hence, we accept H3.

Although ROAA is a measure of financial efficiency that best reflects a bank’s ability to generate profit, ROAE is more important from the investors’ point of view. Table 7 presents the results of the ROAE model estimation for Islamic and conventional banks, the Hausman test indicates that the FE model is appropriate. It should be noted that the models for ROAE most often show lower adjustment to empirical data because of the leverage effect. In the case of our models, the lower adjustment to empirical data is especially visible in the model for Islamic banks. From our point of view, however, more important than R^2^ is the statistical significance of the model parameters, because our study is explanatory and not predictive.

**Table 7:** Determinants of profitability measured by ROAE.

Dependent variable: return on average equity (*ROAE*)		
Explanatory variables	Islamic banks	Conventional banks
*LogTA*	33.528* (19.871)	0.954 (5.296)
*ETA*	0.096 (0.295)	0.325 (0.237)
*NLTA*	-0.336 (0.386)	-0.110 (0.090)
*CI*	-0.118** (0.047)	-0.271** (0.116)
*NPL*	-0.131 (0.081)	-0.289* (0.152)
*GDP*	0.762 (0.654)	0.609*** (0.140)
*CPI*	0.270 (0.609)	0.098 (0.079)
*OP*	-0.003 (0.055)	0.024 (0.018)
*Constant*	-191.688 (136.421)	15.261 (41.116)
Observations	439	791
Number of banks	76	117
Within R^2^	0.08	0.42
Between R^2^	0.03	0.18
Overall R^2^	0.04	0.27
Hausman-test	χ2(8) = 18.26 p-value = 0.02	χ^2^(8) = 50.36 *p*-value = 0.00

Note: This table presents the results of the fixed effect models for Islamic and conventional banks (dependent variable ROAE).

***, **, and * indicate statistical significance at the 1%, 5%, and 10% levels, respectively. Robust standard errors are presented in parentheses.

Considering bank-specific factors, a differentiated impact on ROAE can be observed. There is a weak positive relationship between the size of Islamic banks and return on equity. However, we did not observe a similar relationship for conventional banks. Our findings offer partial empirical support for H4, which suggests that bank size positively affects profitability but only in the case of Islamic banks.

It also appears that asset quality has a stronger impact on the ROAE of conventional banks than that of Islamic banks. Our research indicates that there is a weak negative relationship between *NPL* and ROAE demonstrated by the model for conventional banks. Thus, based on the ROAE as the dependent variable, we can at least partially confirm the correctness of H7.

In the case of both Islamic and conventional banks, we did not notice a significant impact of capital adequacy and liquidity ratios on ROAE. However, there was a strong relationship between *CI* and ROAE for both the Islamic and conventional banks. Hence, based on the ROAE as the dependent variable, we can confirm the correctness of H8.

We also observed differences in the impact of macroeconomic variables on ROAE. There is a clear positive relationship between the change in GDP and the return on equity of conventional banks. This phenomenon did not occur for Islamic banks. We did not observe a clear impact of the remaining macroeconomic variables on the ROAE of both Islamic and conventional banks. Summary of hypotheses verification has been presented in Table 8.

**Table 8:** Hypotheses verification.

Hypothesis	Accepted/Partially accepted/Rejected
H1: GDP growth positively affects the profitability of conventional and Islamic banks.	accepted
H2: There is a positive relationship between the inflation rate and banks profitability for conventional banks and a negative relationship for Islamic banks.	rejected
H3: Oil price increase positively affects only the profitability of Islamic banks.	accepted
H4: Bank size positively affects the profitability of conventional and Islamic banks.	partially accepted
H5: Bank liquidity positively affects the profitability of conventional and Islamic banks.	rejected
H6: Capital adequacy positively affects the profitability of conventional and Islamic banks.	partially accepted
H7: There is a negative relationship between share of non-performing loans in the total gross loans and the profitability of conventional and Islamic banks.	accepted
H8: There is a negative relationship between the cost of running operations divided by operating income and the profitability of conventional and Islamic banks.	accepted

The estimated models show that for Islamic banks operating in the Middle East region, in the analysed period, both the ROAA and ROAE were significantly dependent on two bank-s factors: the size of the bank and the level of operational costs. The size of the bank positively contributes to the profitability of Islamic banks. Since Islamic banks are much younger than conventional banks, they can benefit from economies of scale without suffering from bureaucratic procedures, as is often the case with conventional banks. Therefore, consolidation processess should be encouraged between Islamic banks. When it comes to Islamic banks, similar results were found by Abduh and Idrees [19], Srairi [12], and Al-Qudah and Jaradat [68], whereas Kolapo et al. [66] and Anarfi et. al [67] achieved the same result as we did in relation to conventional banks. However, this result did not confirm that findings of Aladwan [65] and Al-Homaidi et al. [47], who reported a negative and significant relationship between the bank’s size and its profitability. As expected, the empirical findings suggest that efficiency has a negative and significant impact on the performance of both Islamic and conventional banks. Thus, bank managers should focus on controlling overheads and operating costs to improve profitability. This result is consistent with the findings of previous studies (e.g., [7,23,49,70,73].

Additionally, the asset quality ratio reveals negative relationships with the ROAA in both groups of banks, although it is more significant for the conventional banks. Such a result confirms the assumption that an increase in the non-performing loans requires a bank to allocate a significant part of its profit to provisions to cover expected credit losses and as a result profitability will suffer. Similar results were achieved by Ally [72] and Detragiache et al. [73]. Furthermore, the ROAA of Islamic banks was significantly affected by one more bank-specific factor–the capital adequacy ratio. Equity to total assets ratios revealed a positive impact on Islamic banks’ performance. There are several reasons which explain such a result. First, the Islamic banks, whose capital position is strong, can explore business prospects more successfully, which leads to higher profitably. Second, well-capitalised banks are better equipped to control expenses and increase profitability since they have lower needs and can acquire external financing at lower costs. Third, well-capitalised banks will require less borrowing to fund a given amount of assets [25]. Moreover, higher capital adequacy ratio is an important indication of a bank’s creditworthiness and stability [23]. Our results confirmed the findings of Zarrouk et al. [25]. Contradictory, Wasiuzzaman and Tarmizi [58] revealed that the equity to total assets ratio has statistically significant negative effect on the profitability of Islamic banks.

When it comes to macroeconomic factors, the ROAA of both the Islamic and the conventional banks, as well as the ROAE of the conventional banks, was affected by GDP growth. This result is expected and parallel with many other studies (e.g. [44,50,56–58]). In times of economic growth, the quality of the loan portfolio improves, there are fewer credit losses, and less provisions that banks need to retain, which boosts bank profitability. However, a contradictory result was found by Al-Homaidi et al. [54], and Mateev and Bachvarov [51] who found that GDP has a negative impact on the performance of banks. Finally, an important finding of this study is that the ROAA of Islamic banks showed a significantly positive relationship with the price of oil, which may suggest that these banks are still too highly dependent on customers engaged in oil-related business, in contrast to conventional banks, so the decline of oil prices results in their lower profitability. On the other hand, they can use the periods of increased oil prices to make higher profits by extending financing to oil-related businesses. Since Islamic banks rely on profit and loss sharing, this period will be the right time to provide finance. However, since the price of oil is volatile, the banks are advised to provide finance for short periods [89]. Our findings were consistent with the results of studies by Ali et al. [20] and to some extent, Esmaeil et al. [62], whose analysis showed that oil prices significantly affect the profitability of not only Islamic banks but conventional ones as well.

## 7. Robustness checks and alternative specifications

In this section, we performed additional analysis to check the robustness of the previously obtained results. For this purpose, we performed estimation using random effects models. This approach is often found in the literature as a way to test the robustness of results, especially when the signs of the estimated time-varying parameters and their statistical significance are more important for the inference than their exact values [90,91]. The results of the model estimation for the dependent variable ROAA are presented in Table 9, and for the dependent variable ROAE in Table 10.

**Table 9:** Determinants of profitability measured by ROAA, RE estimation.

Dependent variable: return on average assets (*ROAA*)		
Explanatory variables	Islamic banks	Conventional banks
*LogTA*	1.881*** (0.675)	-0.414 (0.345)
*ETA*	0.066** (0.026)	0.057 (0.028)
*NLTA*	0.008 (0.010)	-0.004 (0.009)
*CI*	-0.019* (0.010)	-0.028*** (0.005)
*NPL*	-0.009 (0.016)	-0.032** (0.013)
*GDP*	0.070** (0.033)	0.058*** (0.016)
*CPI*	0.063*** (0.022)	0.007 (0.006)
*OP*	0.008* (0.004)	0.003 (0.002)
*Constant*	-13.022** (5.601)	4.871 (2.872)
Observations	440	791
Number of banks	76	117
Within R^2^	0.37	0.41
Between R^2^	0.07	0.28
Overall R^2^	0.21	0.36

Note: This table presents the results of the random effect models for Islamic and conventional banks (dependent variable ROAA).

***, **, and * indicate statistical significance at the 1%, 5%, and 10% levels, respectively. Robust standard errors are presented in parentheses.

**Table 10:** Determinants of profitability measured by ROAE, RE estimation.

Dependent variable: return on average assets (*ROAE*)		
Explanatory variables	Islamic banks	Conventional banks
*LogTA*	1.076 (4.108)	0.307 (1.213)
*ETA*	-0.157 (0.173)	0.108 (0.103)
*NLTA*	-0.080 (0.095)	-0.102* (0.055)
*CI*	-0.085* (0.047)	-0.226*** (0.080)
*NPL*	-0.024 (0.032)	-0.092 (0.088)
*GDP*	0.585 (0.556)	0.703*** (0.144)
*CPI*	0.596 (0.377)	0.092 (0.070)
*OP*	-0.083 (0.077)	0.020 (0.012)
*Constant*	15.371 (34.97)	18.811 (12.502)
Observations	439	791
Number of banks	76	117
Within R^2^	0.05	0.39
Between R^2^	0.13	0.24
Overall R^2^	0.12	0.31

Note: This table presents the results of the random effect models for Islamic and conventional banks (dependent variable ROAE).

***, **, and * indicate statistical significance at the 1%, 5%, and 10% levels, respectively. Robust standard errors are presented in parentheses.

The results of the random effect models estimated are generally similar to those obtained using fixed effect models. In the case of the ROAA of Islamic banks, only a difference was observed in the impact of inflation. However, we recognise this impact as inconclusive because the other models do not support this observation.

To further test the robustness of the results, we replaced some of the independent variables in the estimated models with alternative indicators. We used net loans divided by the sum of deposits and short-term funding (*NLD*) as a measure of liquidity, non-interest expense divided by average assets (*NIEAA*) as a measure of efficiency, and loan loss reserve to gross loans (*LLRGL*) as a measure of asset quality. The results obtained were presented in S4 and S5 Tables (Supporting information). They are generally qualitatively similar to the results of the original models, but it should be noted that the alternative models have a lower degree of fit to the empirical data, and the alternative independent variables used have lower explanatory properties.

To examine the sensivity of the results, we estimated the model by dividing the sample into subsamples. The first subsample consisted of banks operating in Gulf Cooperation Council (GCC) countries, while the second subsample consisted of countries which are not members of the GCC. The applied division is significant because GCC member states, being the leading oil producers amongst the Middle Eastern countries, strive to create similar or identical regulations in various areas, including economic, financial, trade, customs, and communication regulations, as well as in the educational and cultural spheres. The estimation results of the models for ROAA were presented in Table 11, and for ROAE in Table 12.

**Table 11:** Determinants of profitability measured by ROAA, subsamples analysis.

Dependent variable: return on average assets (*ROAA*)				
Explanatory variables	Islamic banks		Conventional banks	
	GCC countries	Non-GCC countries	GCC countries	Non-GCC countries
*LogTA*	2.264 (1.785)	4.550** (2.216)	-0.726 (0.442)	1.694** (0.705)
*ETA*	0.088*** (0.026)	0.306*** (0.082)	0.100*** (0.031)	0.135*** (0.026)
*NLTA*	0.046* (0.023)	0.003 (0.017)	-0.011 (0.014)	-0.006 (0.011)
*CI*	-0.054*** (0.023)	-0.005 (0.008)	-0.053*** (0.011)	-0.020*** (0.002)
*NPL*	-0.052* (0.028)	-0.005 (0.013)	-0.099*** (0.028)	-0.012* (0.006)
*GDP*	0.056 (0.053)	0.024 (0.018)	0.065*** (0.024)	0.090*** (0.014)
*CPI*	0.056 (0.062)	0.029* (0.014)	-0.065* (0.037)	0.009* (0.005)
*OP*	0.011** (0.004)	0.003 (0.012)	0.004* (0.002)	0.003 (0.002)
*Constant*	-33.688* (18.019)	-34.716** (15.662)	8.239** (3.943)	-10.797** (4.743)
Observations	316	124	428	363
Number of banks	45	31	59	58
Within R^2^	0.62	0.54	0.45	0.69
Between R^2^	0.12	0.16	0.63	0.25
Overall R^2^	0.34	0.19	0.47	0.33
Hausman-test	χ^2^(8) = 95.82 *p*-value = 0.00	χ^2^(8) = 43.61 *p*-value = 0.00	χ^2^(8) = 350.96 *p*-value = 0.00	χ^2^(8) = 75.18 *p*-value = 0.00

Note: This table presents the results of the fixed effect models for Islamic and conventional banks (dependent variable ROAA).

***, **, and * indicate statistical significance at the 1%, 5%, and 10% levels, respectively. Robust standard errors are presented in parentheses.

**Table 12:** Determinants of profitability measured by ROAE, subsamples analysis.

Dependent variable: return on average assets (*ROAE*)				
Explanatory variables	Islamic banks		Conventional banks	
	GCC countries	Non-GCC countries	GCC countries	Non-GCC countries
*LogTA*	5.588*** (1.888)	26.695 (39.197)	-2.703 (5.742)	1.380 (7.040)
*ETA*	0.157** (0.073)	-1.643 (1.462)	0.416 (0.279)	0.363* (0.201)
*NLTA*	0.018 (0.054)	-1.840 (0.054)	-0.141 (0.106)	-0.240 (0.201)
*CI*	-0.137*** (0.020)	-0.252 (0.158)	-0.426*** (0.117)	-0.222* (0.114)
*NPL*	-0.018 (0.021)	-0.185 (0.378)	-0.485* (0.250)	-0.054 (0.071)
*GDP*	0.108 (0.185)	1.296 (1.231)	0.337** (0.137)	1.155*** (0.323)
*CPI*	0.786* (0.470)	0.481 (0.847)	-0.318 (0.240)	-0.160* (0.093)
*OP*	0.012 (0.019)	-0.040 (0.153)	0.037** (0.015)	-0.015 (0.033)
*Constant*	-32.534** (12.721)	-17.252 (304.296)	47.642 (42.065)	13.909 (57.232)
Observations	316	123	428	363
Number of banks	45	31	59	58
Within R^2^	0.32	0.13	0.50	0.45
Between R^2^	0.65	0.04	0.27	0.12
Overall R^2^	0.52	0.03	0.27	0.25
Hausman-test	χ^2^(8) = 34.03 *p*-value = 0.00	χ^2^(8) = 9.10 *p*-value = 0.33	χ^2^(8) = 25.98 *p*-value = 0.00	χ^2^(8) = 21.71 *p*-value = 0.00

Note: This table presents the results of the fixed effect models for Islamic and conventional banks (dependent variable ROAE).

***, **, and * indicate statistical significance at the 1%, 5%, and 10% levels, respectively. Robust standard errors are presented in parentheses.

The results obtained in the subsamples generally confirm the previous conclusions. However, there are some differences in the results for both subsamples. For example, the bank size had a stronger impact on the ROAA of banks in non-GCC countries because banks in this subsample were on average smaller and showed greater diversity, resulting in a clearer impact of size on profitability. The influence of oil prices was significant on the ROAA of Islamic banks in the GCC region. On the other hand, the impact of the GDP variable on ROAA and ROAE was significant only for conventional banks in the subsamples.

## 8. Conclusions

The empirical analysis allows us to shed some light on the relationship between bank-specific factors as well as macroeconomic indicators and the profitability of Islamic and conventional banks in the Middle East. Using a fixed effect panel data analysis on a sample of 270 banks (111 Islamic and 159 conventional banks) from 12 countries over the period 2012–2020, we confirmed some of the findings from earlier studies: for instance, a positive relationship between GDP growth and banks profitability, a negative relationship between cost-income ratio and profitability, and a negative relationship between non-performance loans to gross loans ratio and performance of banks. We also found that three factors impact only the profitability of Islamic banks. These are capital adequacy, the size of the bank and oil price. All three variables are positively related to the performace of Islamic banks.

The results of our study are invaluable for bank managers, regulators, investors and customers. While managers need to assess the results of prior management decisions, bank regulators are concerned about the safety and soundness of the banking system. They monitor the performance of banks in order to identify those banks that may be experiencing serious problems. Without regular performance monitoring, important issues might be missed and result in financial failure. Policymakers and regulators should formulate policies that ensure the resilience of banks against crises, such as the Covid-19 pandemic. When comparing conventional and Islamic banks, several noticeable differences in financial indicators and their impact on profitability become evident. Therefore, regulators should not treat the two types of banks identically when creating and implementing banking regulations. For instance, they should apply different incentives for consolidation processes. In the case of conventional banks, further consolidation processes may have negative consequences for customers, competition, and systemic risk. In contrast, the situation is different for Islamic banks, where there are many very small entities and consolidation processes could have a positive impact on their profitability, without having severe negative consequences for customers. Another issue for regulators and bank managers is to lower operational costs. Digital transformation can enhance efficiency, flexibility, and resilience in banking operations for both conventional and Islamic banks. The push towards electronic banking was further propelled by the COVID-19 pandemic. It is also recommended that regulators and policymakers, when making decisions that affect the economy, take into account that some of the macroeconomic factors i.e. GDP growth, affect the profitability of banks.

According to our estimations, oil prices affect only the profitability of Islamic banks. When the price of oil is high, Islamic banks could consider extending financing to oil-related businesses in order to increase profitability. However, since the price of oil is volatile, they are advised to provide mainly short-term financing. They also need to find tools to mitigate any unfavourable influence of oil price shocks.

The empirical findings of our study suggests that both conventional and Islamic banks should focus on improving the process of monitoring non-performing loans, as the negative impact of non-performing loans on profitability has been observed. Banking managers may also undertake measures to reduce exposure to unfavorable sectors and direct attention to more promising industries. Islamic banks still need to find ways to boost non-interest income, while commercial banks should note that a more conservative philosophy towards banking as exemplified by Islamic principles may serve them to avoid losses.

Our study also has some limitations. The main one is some gaps in data on non-performing loans of Islamic banks. However, the available data was numerous enough to determine the impact of bad loans on the performance of both Islamic and conventional banks. Thanks to the large sample size, we consider our research sound and very reliable. Furthermore, in our study we used two variables which measure the profitability of the banking sector ROAA and ROAE. There are other variables which are commonly used to measure profitability. Future work could include i.e. the Net Interest Margin (NIM) and Net Profit Margin (NPM) as a dependent variable for conventional and Islamic banks respectively.

It is recommended that the results of this paper are confirmed against another sample from different regions. Further, it is also recommended that a comparison of Islamic and conventional bank performance does not just consider profitability. This research could be extended by adding new independent variables and by comparing profitability factors between regions. This could also be combined with the approach of Islamic bank stability during the next years of the COVID-19 crisis, including an increase of oil price since it is frequently argued that the resistance towards crisis is due to the distinctive Islamic bank investment policies.

## Supporting information

S1 Table Pairwise correlation and multicollinearity diagnostics for Islamic banks. (TIF)

S2 Table Pairwise correlation and multicollinearity diagnostics for conventional banks. (TIF)

S3 Table Results of dynamic models estimation for Islamic and conventional banks. (TIF)

S4 Table Determinants of profitability measured by ROAA using alternative independent variables. (TIF)

S5 Table Determinants of profitability measured by ROAE using alternative independent variables. (TIF)

S1 Data (XLSX)
