# Supplementary figures and images for: Is the profitability of Islamic and conventional banks driven by the same factors?—A study of banking in the Middle East
Source: PLoS One. 2023 Aug 7;18(8):e0289264. doi: 10.1371/journal.pone.0289264 (PMC10406290; doi:10.1371/journal.pone.0289264)

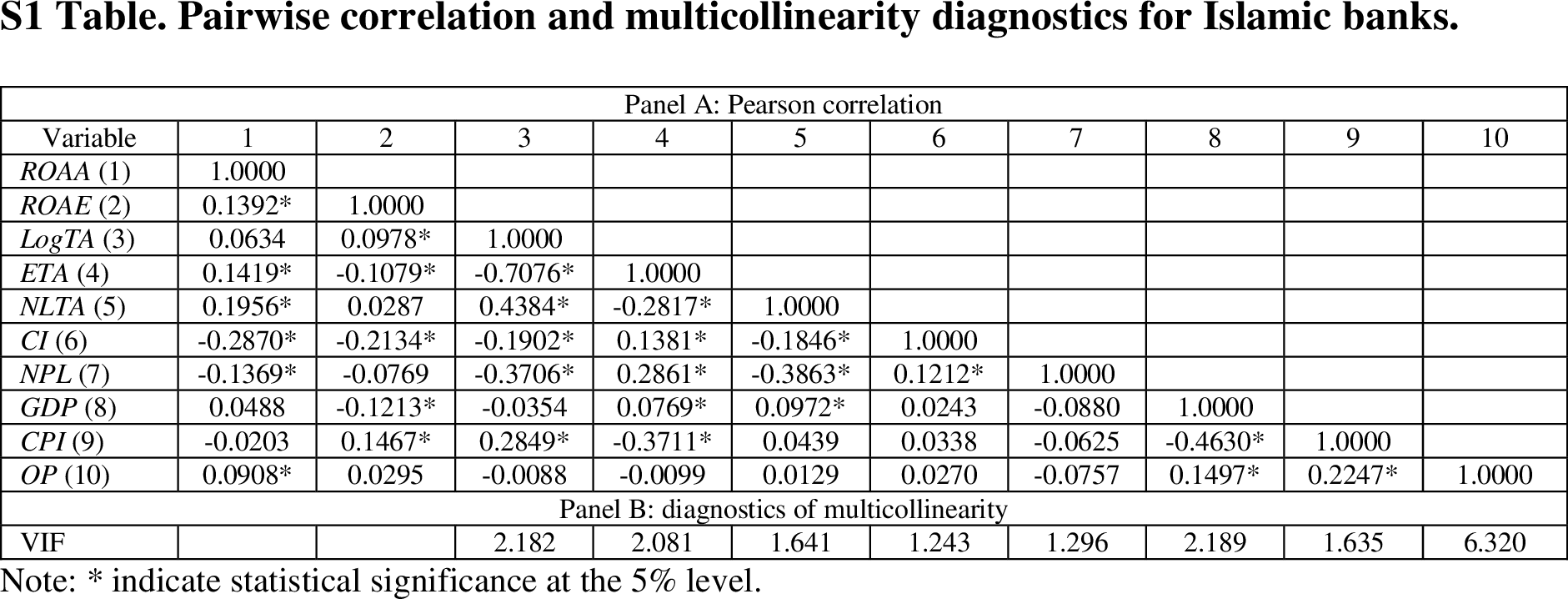

Supplement: S1 Table — (TIF) [file pone.0289264.s001.tif]

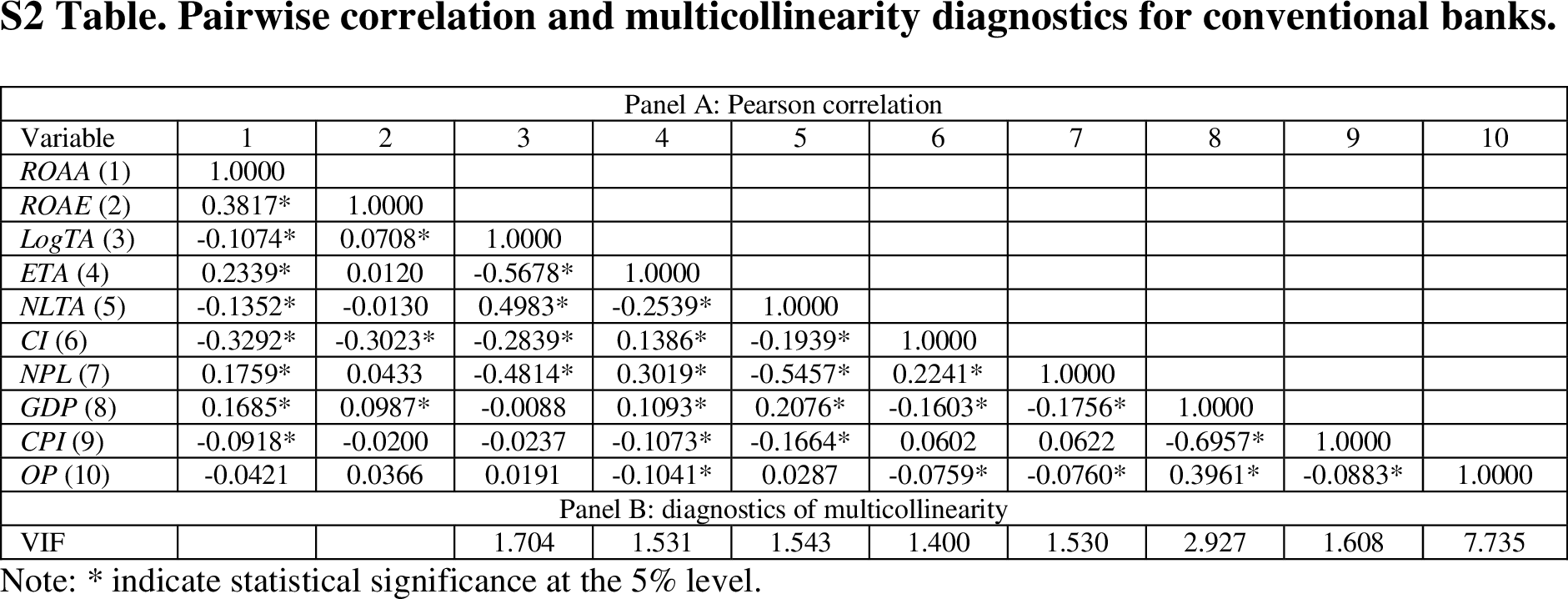

Supplement: S2 Table — (TIF) [file pone.0289264.s002.tif]

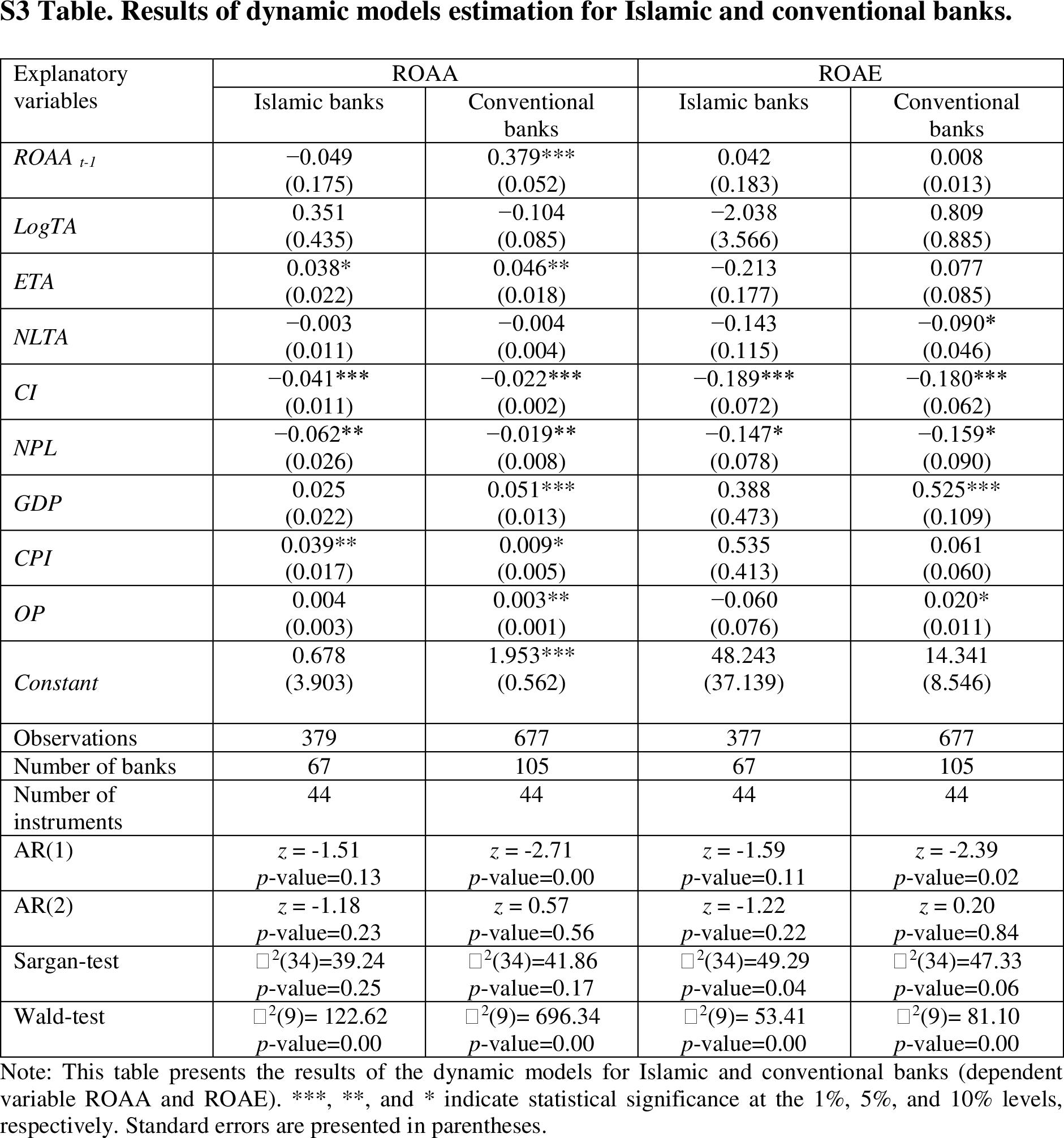

Supplement: S3 Table — (TIF) [file pone.0289264.s003.tif]

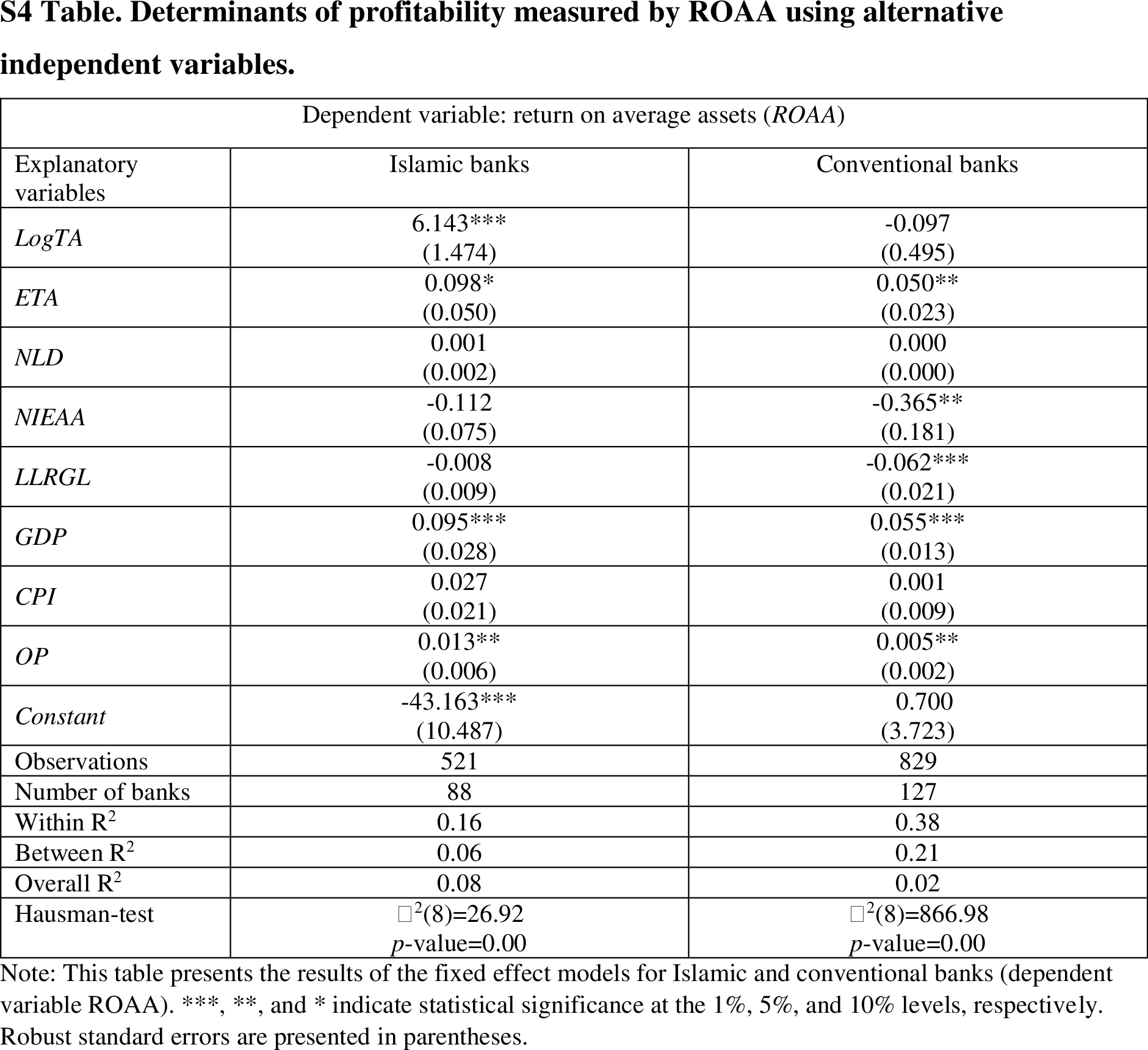

Supplement: S4 Table — (TIF) [file pone.0289264.s004.tif]

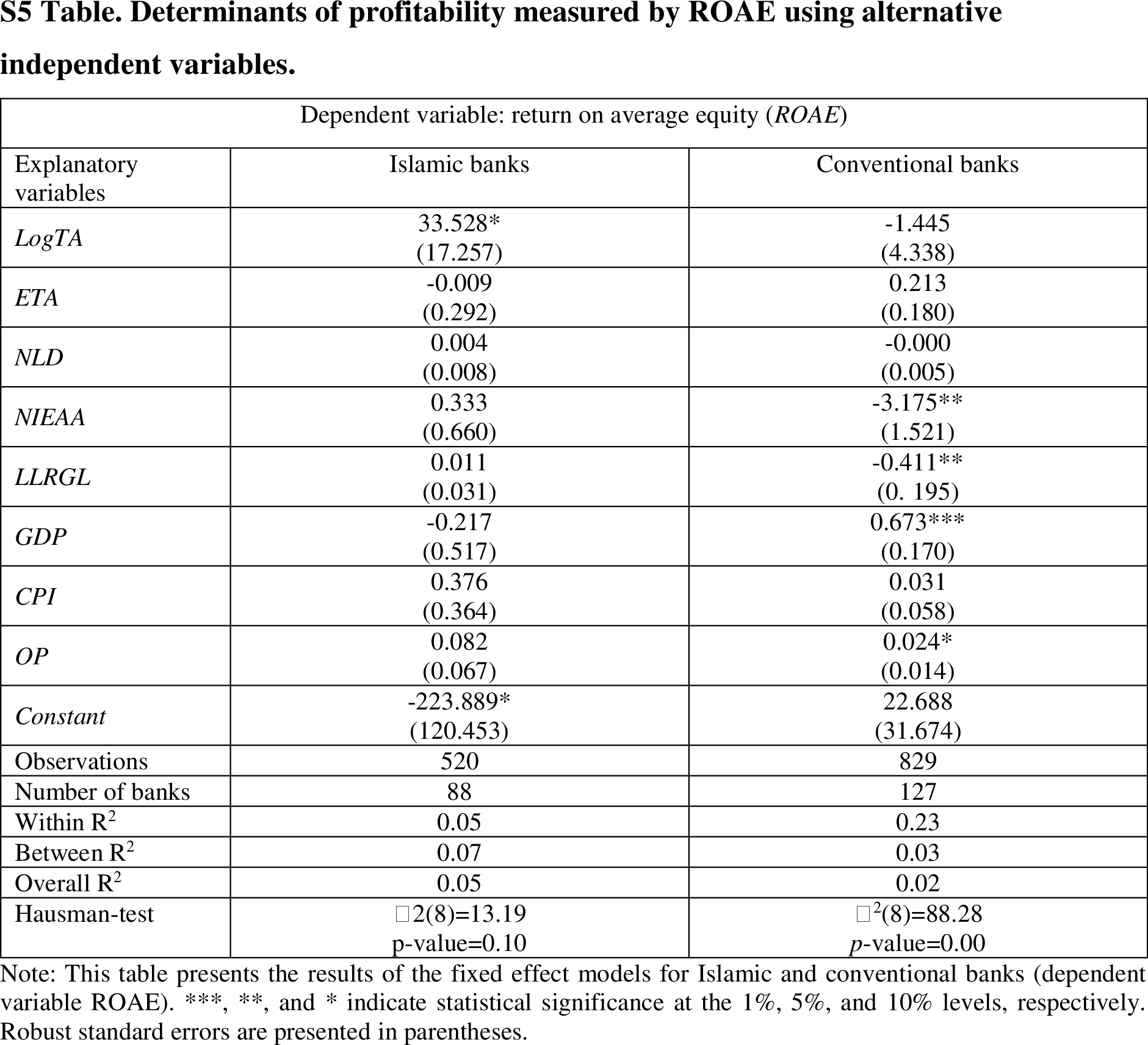

Supplement: S5 Table — (TIF) [file pone.0289264.s005.tif]
